# Association between different types and characteristics of fetal deceleration during labour and neonatal acidemia at delivery: A case-control study

**DOI:** 10.1016/j.eurox.2025.100389

**Published:** 2025-04-25

**Authors:** Maria Fogelberg, Charlotte Dahlbäck, Frida Ekengård, Gisela Rickle, Andreas Herbst

**Affiliations:** a Department of Obstetrics and Gynecology, Skane University Hospital, Sweden; b Institution for Clinical Sciences Lund, Lund University, Sweden; c Institution for Clinical Sciences Malmö, Lund University, Sweden

**Keywords:** Cardiotocography, electronic fetal monitoring, fetal heart rate, decelerations, acidemia

## Abstract

**Objective:**

Classification of fetal heart rate (FHR) decelerations as suspicious or pathological differs between current interpretation templates for intrapartum cardiotocography. Decelerations are the most frequent FHR pattern irregularities during labour. The aim of this study was to assess the association between different types and characteristics of decelerations and acidemia at birth.

**Methods:**

This case-control study includes 365 cases with cord pH < 7.10 after 1st stage cesarean delivery or pH < 7.05 after vaginal delivery at > 34 + 0 weeks after induced or spontaneous labour, and 730 controls with pH ≥ 7.15. Cardiotocographic recordings from 60 min before birth were scrutinized and decelerations evaluated in detail. Odds ratios (OR) with 95 % confidence intervals for acidemia at birth were determined.

**Results:**

The following types of decelerations were associated with acidemia: Late decelerations; OR 9.0 (6.1–13) if > 5, and OR 19 (9.7–37) if repetitive > 20 min, combined decelerations; OR 4.2 (2.7–6.4) if > 5 and OR 6.4 (3.1–13) if repetitive > 20 min, one prolonged deceleration > 5 min; OR 12 (7.9–19), three prolonged decelerations for 3–5 min; OR 10 (4.3–25), and > 5 variable decelerations > 60 s; OR 2.2 (1.6–2.9). For variable decelerations > 60 s, absent variability within decelerations was the only additional characteristic significantly associated with acidemia; OR 5.8 (2.1–16). A strong association with acidemia was noted for a FHR below the baseline ≥ 50 % of the time > 30 min; OR 14 (10−19).

**Conclusion:**

Late and prolonged decelerations are strongly, and combined decelerations moderately associated with acidemia. The risk of acidemia is highly increased if FHR is below baseline ≥ 50 % of the time.

## Highlights

• Increased risk of acidosis with late, combined and prolonged decelerations.
• Risk of acidosis if fetal heart rate is 50 % below baseline for 30 min.
• Only two “concerning criteria” for variable decelerations were associated with acidemia: duration >60 s and loss of variability within decelerations.

## 1 Introduction

The purpose of cardiotocographic (CTG) monitoring during labour is to identify signs of fetal hypoxia before asphyxia ensues. Different guidelines with interpretation templates serve as decision aid for midwives and obstetricians [1], [2], [3], [4]. Since different CTG classification templates are restricted by either low sensitivity or low specificity [5], [6], [7], [8], efforts to improve the diagnostic precision are motivated.

One difference between current classification systems is that the fetal heart rate decelerations resulting in classification pathological differ [1], [2], [3], [4]. For example, according to the ACOG criteria, recurrent variable decelerations are classified as a category III pattern, whereas in the FIGO guidelines (2015) the presence of repetitive variable decelerations only results in a suspicious pattern. In the NICE guidelines (2022), a CTG pattern is classified as pathological if repetitive variable decelerations show additional concerning criteria for > 30 min.

The aim of this study was to determine the associations between acidemia at birth and fetal heart rate decelerations of different types and characteristics during labour. Our intention was to assess for which types of decelerations the association to acidemia is sufficiently high to motivate such a pattern to be included as “pathological” in a classification template.

The present study focused on FHR decelerations and only took other FHR variables into account when integrated in the assessment of variable decelerations in present classification templates, and in performing a sub-analysis including only traces with normal baseline and variability.

## 2 Methods

This is a retrospective case-control study including 365 cases with acidemia at birth and 730 controls. The studied CTG traces derive from births at Skåne University Hospital and Helsingborg Hospital in Region Skåne between 2012–2017. Part of the material has been used in previous studies [7], [8]. The material included singleton pregnancies with spontaneous or induced labour at > 34 completed gestational weeks. For inclusion, ≥ 30 min CTG recording before birth was required with a maximum gap of 30 min between end of recording and birth. A maximum of 10 min of non-interpretable CTG trace (due to poor contact, sequences of maternal heart rate or disconnected CTG) was accepted. All neonates born with acidemia during this period who met the inclusion criteria were included in the study.

In this study, acidemia was defined as cord artery or vein pH < 7.10 after cesarean delivery in the first stage of labour and pH < 7.05 after vaginal birth or second stage cesarean. The higher pH cut-off for the first stage of labour was motivated by the normal decrease of pH during the second stage of labour [9]. Severe acidosis at birth is often defined as pH < 7.0, but recent studies have shown that also pH between 7.00 and 7.19 and particularly pH < 7.10 increases the risk of adverse neonatal outcomes [10], [11]. Cord samples were taken immediately after birth by puncture of the intact cord, except in the case of cesarean section where the umbilical cord was clamped before samples were taken.

Controls were the first two eligible neonates born subsequently after each case in the same hospital. To be eligible as controls, umbilical artery and vein sampling needed to show an umbilical artery pH > 7.15 with an arterio-venous difference > 0.02, and Apgar scores 9 or 10 at five and ten min. Controls had to have > 30 min of CTG recording at the same cervical dilation as the trace from the corresponding case.

Totally 1095 CTG recordings were assessed during 2023–2025; 365 cases with acidemia and 730 controls. In the case group, 70 were delivered by cesarean in the first stage of labour, 24 by cesarean in the second stage, and 271 vaginally. Background data are presented in Table 1.

**Table 1:** Summary of background data of cases and controls.

	Cases n (%)	Controls n (%)	Missing data n	Total n
Total	364	728		1092
Primipara	225 (61.0)	356 (48.6)	0	1092
Instrumental delivery	88 (24.2)	45 (6.2)	0	1092
Cesarean section	94 (25.8)	12(1.6)	0	1092
Breech	2 (0.5)	1(0.1)	0	1092
Preterm birth < 37 + 0	8 (2.2)	18 (2.5)	0	1092
Post-term birth ≥ 42 + 0	28 (7.7)	50 (6.9)	0	1092
Birthweight < 2.5 kg	5 (1.4)	10 (1.4)	3	1089
Birthweight > 4.5 kg	13 (3.6)	18 (2.5)	3	1089
Epidural	159 (43.7)	215 (29.5)	0	1092
Oxytocin augmentation	202 (55.5)	252 (34.6)	0	1092
Fever/Infection	11 (3.0)	8 (1.1)	0	1092
Meconium stained amniotic fluid	96 (26.4)	137 (18.8)	4	1088
Diabetes	22 (6.0)	19 (2.6)	0	1092
Preeclampsia	15 (4.1)	16 (2.2)	0	1092
BMI < 25	209 (57.4)	433 (59.5)	29	1063
BMI > 30	47 (12.9)	80 (11.0)	29	1063
Smoking	20 (5.5)	54 (7.4)	18	1075
5-minute Apgar score < 7	47 (12.9)	0	1	1091
Cord artery pH available	344 (94.5)	728 (100)	20	1072
Cord vein pH available	344 (94.5)	728 (100)	20	1072
Both artery +vein pH available	304 (83.5)	728 (100)	40	1052
Cord artery or vein pH < 7.00	104 (28.6)	0	0	1092
Cord artery or vein pH < 7.05	328 (90.1)	0	0	1092
Cord artery or vein base deficit > 12 mmol/L	96 (26.7)	2 (0.3)	13	1079

The CTG traces from the last hour before birth for cases and the corresponding period for controls were scrutinized and different decelerations were evaluated in detail categorizing each deceleration by type and characteristics. The total number of each type was assessed, as well as repetitivity (occurrence at ≥50 % of contractions).

Decelerations were defined as temporary slowing of the fetal heart rate (FHR) of > 15 beats per minute (bpm) below the baseline, except for late decelerations in a trace with reduced variability where an amplitude of 10 bpm was sufficient.

Early decelerations were defined as uniform periodic slowing of FHR with gradual onset before the contraction peak, ≥ 30 s from beginning to nadir, nadir within 20 s after the contraction peak, and gradual recovery. Late decelerations were defined as uniform periodic slowing of FHR with onset after mid of the contraction and nadir > 20 s after the contraction peak and ending after the contraction [1], [2].

Variable decelerations were defined as intermittent slowing of FHR with rapid onset, less than 30 s from beginning to nadir. Duration was determined as time from start of deceleration (pre-accelerations not included) to return to baseline. They were stratified into two groups depending on duration: ≤ 60 s or > 60 s. The following characteristics were also evaluated: amplitude ≥ 60 bpm, nadir below 60 bpm, absent variability within decelerations, overshoot (exaggerated elevation of the FHR immediately after the deceleration), slow (>2 min) or incomplete return to baseline, loss of previous shouldering and baseline > 160 bpm or variability < 5 bpm between decelerations.

When decelerations had rapid onset but repeatedly had the timing described above for late decelerations, these were classified as late decelerations.

**Fig. 1:**
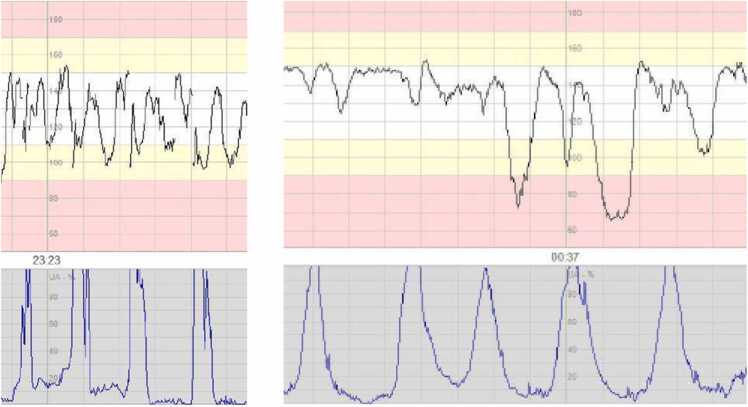
Combined decelerations, defined in study as two decelerations per contraction, occurring repeatedly with complete or incomplete recovery of the fetal heart rate baseline between the two decelerations.

Combined decelerations were defined as two decelerations identified per contraction, occurring repeatedly with complete or incomplete recovery of the baseline fetal heart rate between the two decelerations (Fig. 1). This is typically a combination of variable and late decelerations [4], [12].

Prolonged decelerations were divided, based on duration, into two groups, 3–5 min or > 5 min. Prolonged decelerations > 5 min were stratified into occurring only during the last 10 min before vaginal birth or occurring (beginning) earlier.

**Fig. 2:**
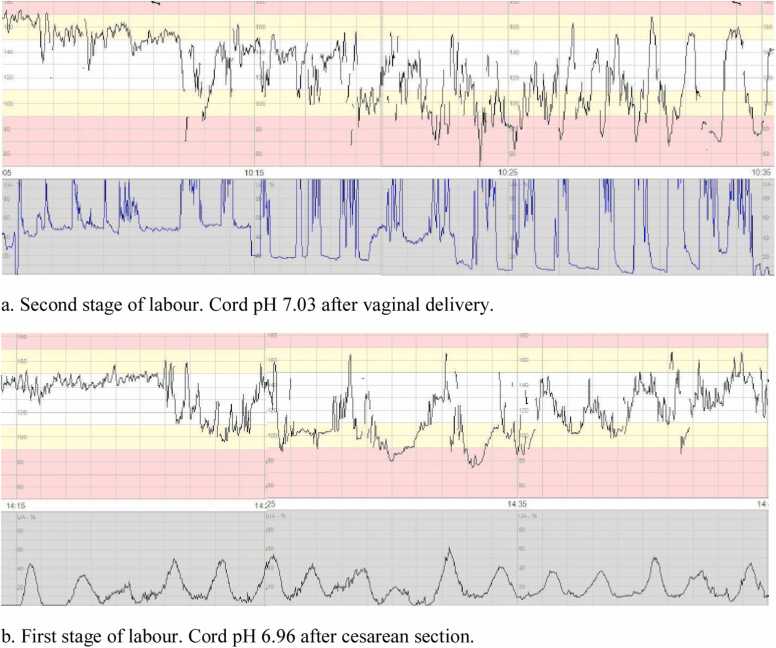
Examples of CTG with fetal heart rate below the baseline ≥ 50 % of the time, due to decelerations.

We also evaluated if the FHR, due to decelerations, was below the baseline ≥ 50 % of the time for more than 30 min (Fig. 2). When assessing this, a stable baseline was first identified, then the number of min that the FHR was below this baseline, due to decelerations, and the number of min above or at the baseline were counted.

All CTG traces were independently scrutinized by two of the authors (MF, AH). When the assessors did not agree regarding the type or number of decelerations, a further assessment was made by a third assessor (CD, FE, GSR). The majority assessment of type and median assessment of number of decelerations was used for the final classification.

The association between each type of deceleration and acidemia at birth was analysed. In a sub-analysis, these associations were determined for traces with normal baseline (110−160) and variability (5–25 bpm).

## 3 Statistical analyses

Statistical analyses were performed using MedCalc for Windows, version 23.1.7 (MedCalc Software, Ostend, Belgium). Odds ratios (OR) with 95 % confidence intervals were calculated for the association of different fetal heart rate patterns to acidosis. For the categorical comparisons, a chi-square test was used for calculation of statistical significance. Since multiple comparisons were made, only differences with a p-value less than 0.01 were considered statistically significant. Significant associations with OR > 10 was considered strong, OR 5–9 as moderate, and OR below 5 as weak.

## 4 Results

Of the 1095 studied cardiotocographic recordings, one case was excluded due to registration of maternal pulse instead of the FHR. The corresponding two controls were also excluded, leaving 1092 CTG traces for analysis. A full assessment of 60 min was met by 77 % of CTGs. The mean was 56 min.

Table 2 presents associations between acidemia at birth and decelerations of different characteristics, during (30-) 60 min in the first and second stage of labour. Neither early uniform decelerations nor variable decelerations < 60 s were associated with acidemia.

**Table 2:** Association between acidemia at birth and different types of decelerations in the first and second stage of labour. Figures are presented as numbers (%) and odds ratios with 95 % confidence intervals.

	1st stage				2nd stage				1st and 2nd stage			
	Acidemia N = 70	Controls N = 140	OR (95 % CI)	p-value	Acidemia N = 294	Controls N = 588	OR (95 % CI)	p-value	Acidemia N = 364	Controls N = 728	OR (95 % CI)	p-value
**Early decelerations**												
< 5	65 (92.9)	130 (92.9)	*Reference*		293 (99.7)	576 (98.0)	*Reference*		358 (98.4)	706 (97.0)	*Reference*	
≥ 5	5 (7.1)	10 (7.1)	1.0 (0.3–3.0)	p = 1.000	1 (0.3)	12 (2.0)	0.2 (0.02–1.3)	p = 0.083	6 (1.6)	22 (3.0)	0.5 (0.2–1.3)	P = 0.18
**Late decelerations**												
< 5	42 (60.0)	139 (99.3)	*Reference*		193 (65.6)	547 (93.0)	*Reference*		235 (64.6)	686 (94.2)	*Reference*	
5–9	15 (21.4)	0	102 (6.0–1736)	p = 0.001	47 (16.0)	28 (4.8)	4.8 (2.9–7.8)	p < 0.0001	62 (17.0)	28 (3.8)	6.5 (4.0–10)	p < 0.0001
≥ 10	13 (18.6)	1 (0.7)	43 (5.5–339)	p = 0.0004	54 (18.4)	13 (2.2)	12 (6.3–22)	p < 0.0001	67 (18.4)	14 (1.9)	14 (7.7–25)	p < 0.0001
≥ 5	28 (40.0)	1 (0.7)	93 (12–702)	p < 0.0001	101 (34.4)	41 (7.0)	7.0 (4.7–10)	p < 0.0001	129 (35.4)	42 (5.8)	9.0 (6.1–13)	p < 0.0001
Repetitive > 20 min												
No	49 (70.0)	140 (100)	*Reference*		239 (81.3)	578 (98.3)	*Reference*		288 (79.1)	718 (98.6)	*Reference*	
Yes	21 (30.0)	0	122 (7.2–2053)	p = 0.0008	55 (18.7)	10 (1.7)	13 (6.7–27)	p < 0.0001	76 (20.9)	10 (1.4)	19 (9.7–37)	p < 0.0001
**Combined decelerations**												
< 5	59 (84.3)	140 (100)	*Reference*		240 (81.6)	551 (93.7)	*Reference*		299 (82.1)	691 (94.9)	*Reference*	
5–9	9 (12.9)	0	45 (2.6–783)	p = 0.009	28 (9.5)	29 (4.9)	2.2 (1.3–3.8)		37 (10.2)	29 (4.0)	2.9 (1.8–4.9)	p < 0.0001
≥ 10	2 (2.9)	0	12 (0.6–250)	p = 0.11	26 (8.8)	8 (1.4)	7.5 (3.3–17)	p < 0.0001	28 (7.7)	8 (1.1)	8.1 (3.6–18)	p < 0.0001
≥ 5	11 (15.7)	0	54 (3.2–937)	p = 0.006	54 (18.4)	36 (6.1)	3.4 (2.2–5.4)	p < 0.0001	65 (17.9)	36 (4.9)	4.2 (2.7–6.4)	p < 0.0001
Repetitive > 20 min												
No	66 (94.3)	140 (100)	Reference		268 (91.2)	578 (98.2)	*Reference*		334 (91.8)	718 (98.6)	*Reference*	
Yes	4 (5.7)	0	19 (1.01–358)	p = 0.049	26 (8.8)	10 (1.7)	5.6 (2.7–12)	p < 0.0001	30 (8.2)	10 (1.4)	6.4 (3.1–13)	p < 0.0001
**Variable decelerations < 60 s**												
< 5	51 (72.9)	119 (85.0)	*Reference*		108 (36.7)	170 (28.9)	*Reference*		159 (43.7)	289 (39.7)	*Reference*	
5–9	14 (20.0)	15 (10.7)	2.2 (1.0–4.8)	p = 0.056	75 (25.5)	190 (32.3)	0.6 (0.4–0.9)	p = 0.0096	89 (24.5)	205 (28.2)	0.8 (0.6–1.1)	P = 0.14
≥ 10	5 (7.1)	6 (4.3)	1.9 (0.6–6.7)	p = 0.28	111 (37.8)	228 (38.8)	0.8 (0.6–1.1)	p = 0.11	116 (31.9)	234 (32.1)	0.9 (0.7–1.2)	P = 0.49
≥ 5	19 (27.1)	21 (15.0)	2.1 (1.0–4.3)	p = 0.036	186 (63.3)	418 (71.1)	0.7 (0.5–0.9)	p = 0.018	205 (56.3)	439 (60.3)	0.8 (0.7–1.1)	P = 0.21
**Variable decelerations > 60 s**												
< 5	63 (90.0)	140 (100)	*Reference*		189 (64.3)	464 (78.9)	*Reference*		252 (69.2)	604 (83.0)	*Reference*	
5–9	6 (8.6)	0	29 (1.6–518)	p = 0.022	78 (26.5)	99 (16.8)	1.9 (1.4–2.7)	p = 0.0002	84 (23.0)	99 (13.6)	2.0 (1.5–2.8)	p < 0.0001
≥ 10	1 (1.4)	0	6.6 (0.3–165)	p = 0.25	27 (9.2)	25 (4.3)	2.7 (1.5–4.7)	p = 0.0008	28 (7.7)	25 (3.4)	2.7 (1.5–4.7)	p = 0.0003
≥ 5	7 (10.0)	0	33 (1.9–590)	p = 0.017	105 (35.7)	124 (21.1)	2.1 (1.5–2.8)	p < 0.0001	112 (30.8)	124 (17.0)	2.2 (1.6–2.9)	p < 0.0001
Repetitive ≥ 30 min												
No	69 (98.7)	140 (100)	*Reference*		272 (92.5)	560 (95.2)	*Reference*		341 (93.7)	700 (96.1)	Reference	
Yes	1 (1.4)	0	6.1 (0.2–150)	p = 0.27	22 (7.5)	28 (4.8)	1.6 (0.9–2.9)	p = 0.10	23 (6.3)	28 (3.8)	1.7 (1.0–3.0)	p = 0.0707
**Prolonged decelerations ≥ 5 min**												
0	41 (58.6)	138 (98.6)	*Reference*		185 (62.9)	517 (87.9)	*Reference*		226 (62.1)	655 (90.0)	*Reference*	
≥ 1 only last 10 min	-	-	-	-	23 (7.8)	46 (7.8)	1.4 (0.8–2.4)	p = 0.21	23 (6.3)	46 (6.3)	1.4 (0.9–2.4)	p = 0.16
≥ 1 before last 10 min	29 (41.4)	2 (1.4)	49 (11–213)	p < 0.0001	86 (29.2)	25 (4.3)	9.6 (6.0–15)	p < 0.0001	115 (31.6)	27 (3.7)	12 (7.9–19)	p < 0.0001
**Prolonged decelerations 3–5 min**												
< 3	68 (97.1)	139 (99.3)	*Reference*		267 (90.8)	583 (99.1)	*Reference*		335 (92.0)	722 (99.2)	*Reference*	
≥ 3	2 (2.9)	1 (0.7)	4.1 (0.4–46)	p = 0.25	27 (9.2)	5 (0.9)	12 (4.5–31)	p < 0.0001	29 (8.0)	6 (0.8)	10 (4.3–25)	p < 0.0001
**≥ 50 % below the baseline**												
No	42 (60.0)	140 (100)	*Reference*		95 (32.3)	510 (86.7)	*Reference*		137 (37.6)	650 (89.3)	*Reference*	
Yes	28 (40.0)	0	188 (11–3152)	p = 0.0003	199 (67.7)	78 (13.3)	14 (9.7–19)	p < 0.0001	227 (62.4)	78 (10.7)	14 (10-19)	p < 0.0001

Late decelerations were strongly associated with acidemia; OR 14 (7.7–25) when ten or more were identified, and 19 (9.7–37) if they were repetitive > 20 min.

Variable decelerations > 60 s were weakly associated with acidemia with an OR 2.7 (1.5–4.7) when > 10 were identified. For repetitive variable decelerations no significant association with acidemia was found.

For combined decelerations the association to acidemia was moderate with OR 8.1 (3.6–18) for a count > 10, and OR 6.4 (3.1–13) if they were repetitive for 20 min.

Associations with acidemia were strong for one prolonged deceleration > 5 min (OR 12; 7.9–19), unless occurring last 10 min before birth, and for three or more prolonged decelerations of 3–5 min (OR 10; 4.3–25).

Fetal heart rate below the baseline ≥ 50 % of the time for more than 30 min was strongly associated with acidemia (OR 14; 10–19).

Most deceleration patterns associated with acidemia were considerably less frequent in controls during the first than in the second stage, resulting in higher OR estimates in the first stage. In the first stage only one control had five late decelerations, none had five variable decelerations > 60 s or combined decelerations, and three had prolonged decelerations.

For decelerations associated with acidemia at birth, we performed a sub-analysis including only traces with normal baseline and variability (Table 3). Significant associations to acidemia remained for late decelerations, combined decelerations, variable decelerations > 60 s, prolonged decelerations and FHR 50 % below baseline.

**Table 3:** Association between different type of decelerations and acidemia at birth, in fetal heart rate traces with baseline 110–160 and variability 5–25 bpm.

	Cases with acidemia (%)	Controls (%)	OR (95 % CI)	P-value
	**N = 204**	**N =661**		
**Late decelerations**				
< 5	143 (70.1)	626 (94.7)	reference	
> 5	61 (29.9)	35 (5.3)	7.6 (4.8-12)	>0.0001
5–9	35 (17.1)	24 (3.6)	6.4 (3.7–11)	<0.0001
> 10	26 (12.7)	11 (1.7)	10 (5.0–21)	<0.0001
Not repetitive > 20 min	172 (84.3)	651 (98.5)	reference	
Repetitive > 20 min	32 (15.7)	10 (1.5)	12 (5.8–25)	<0.0001
**Combined decelerations**				
< 5	159 (77.9)	630 (95.5)	reference	
> 5	45 (22.1)	30 (4.5)	5.9 (3.6-9.7)	<0.0001
5–9	25 (12.3)	25 (3.8)	4.0 (2.2–7.1)	<0.0001
> 10	20 (9.8)	6 (0.9)	13 (5.2–33)	<0.0001
Not repetitive > 20 min	182 (89.2)	653 (98.8)	reference	
Repetitive > 20 min	22 (10.8)	8 (1.2)	9.9 (4.3–23)	<0.0001
**Variable decelerations > 60 seconds**				
< 5	129 (63.2)	551 (83.4)	reference	
> 5	75 (36.8)	110 (16.6)	2.9 (2.0–4.1)	<0.0001
5–9	55 (27.0)	90 (13.6)	2.6 (1.8–3.8)	<0.0001
> 10	20 (9.8)	20 (3.0)	4.3 (2.2–8.2)	<0.0001
Not repetitive > 30 min	189 (92.6)	639 (96.7)	reference	
Repetitive > 30 min	15 (7.4)	22 (3.3)	2.3 (1.2–4.5)	0.01
**Prolonged deceleration(s) 3–5min**				
< 3	185 (90.7)	656 (99.2)	reference	
> 3	19 (9.3)	5 (0.8)	13 (5.0–37)	<0.0001
**Prolonged deceleration(s) > 5min**				
None	139 (68.1)	599 (90.6)	reference	
Last 10 min before birth	14 (6.9)	43 (6.5)	1.4 (0.75–2.6)	0.29
Before last 10 min	51 (25.0)	19 (2.8)	12 (6.6–20)	<0.0001
**Fetal heart rate > 50 % below baseline during > 30min**				
No	79 (38.7)	595 (90.0)	reference	
Yes	125 (61.3)	66 (10.0)	14 (9.8–21)	<0.0001

For variable decelerations > 60 s, a further analysis was performed comparing those with and without additional potentially concerning characteristics (Table 4). Of the examined characteristics, loss of variability within decelerations was significantly associated with acidemia (OR 5.8; 2.1–16), whereas amplitude > 60 beats, nadir below 60 bpm, failed return to baseline, duration > 2 min (slow return), loss of shouldering, overshoot and baseline > 160 bpm or variability < 5 bpm between the decelerations were not.

**Table 4:** Association between other potentially concerning characteristics of variable decelerations with duration > 60 seconds and acidemia at birth. Only traces with > 5 variable decelerations were included, and each potentially concerning factor had to be present in > 3 decelerations to be accounted for.

	**Acidemia (%)**	**Controls (%)**	**OR (95 % CI)**	**P-value**
	**N = 112**	**N = 124**		
**Amplitude > 60 bpm**				
No	41 (36.6)	54 (43.5)	*reference*	
Yes	71 (63.4)	70 (56.5)	1.3 (0.79–2.3)	0.28

**Nadir at < 60 bpm**				
No	96 (85.7)	104 (83.9)	*reference*	
Yes	16 (14.3)	20 (16.1)	0.87 (0.42–1.8)	0.69

**Slow or failed return to baseline**				
No	74 (66.1)	90 (72.6)	*reference*	
Yes	38 (33.9)	34 (27.4)	1.4 (0.78–2.4)	0.28

**Loss of previously present shouldering**				
No	111 (99.1)	120 (96.8)	*reference*	
Yes	1 (0.8)	4 (3.2)	0.27 (0.03–2.5)	0.25

**Absent variability within decelerations**				
No	90 (80.4)	119 (96.0)	*reference*	
Yes	22 (19.6)	5 (4.0)	5.8 (2.1–16)	0.0006

**Overshoot**				
No	110 (98.2)	123 (99.2)	*reference*	
Yes	2 (1.8)	1 (0.8)	2.2 (0.20–25)	0.51

**Baseline > 160 between decelerations**				
No	98 (87.5)	117 (94.4)	*reference*	
Yes	14 (12.5)	7 (5.6)	2.4 (0.93–6.2)	0.07

**Variability < 5 between decelerations**				
No	106 (94.6)	119 (96.0)	*reference*	
Yes	6 (5.4)	5 (4.0)	1.3 (0.40–4.5)	0.63

For NICE-22 concerning criteria for variable decelerations, we found a significant association for both amber, OR 2.3 (1.5–3.3) and red criteria, OR 3.9 (2.5–5.9) patterns and acidemia at birth (Table 5). For SWE17 we found a significant association for variable decelerations > 60 s with concomitant tachycardia or reduced variability, OR 3.3 (1.5–7.5).

**Table 5:** The associations between amber/suspicious and red/pathological criteria for variable declarations according to NICE −22 and SWE-17 and acidemia at birth.

	**Acidemia (%)**	**Controls (%)**	**OR (95 % CI)**	**P-value**
	**N = 364**	**N = 728**		
**Variable decelerations with concerning characteristics (NICE−22)**				
No	244 (67.0)	622 (85.4)	*reference*	
Repetitive < 30 min or non-repetitive > 30 min	58 (16.0)	65 (9.0)	2.3 (1.5–3.3)	<0.0001
Repetitive > 30 min	62 (17.0)	41 (5.6)	3.9 (2.5–5.9)	<0.0001

**Repetitive variable decelerations > 60 s**				
No	332 (91.2)	695 (95.5)	*reference*	
Yes, with normal baseline and variability	16 (4.4)	23 (3.1)	1.5 (0.76–2.8)	0.26
Yes, with baseline > 160 bpm or variability < 5 bpm	16 (4.4)	10 (1.4)	3.3 (1.5–7.5)	0.003

## 5 Discussion

Since acidemia at birth is a relatively rare event, the positive predictive value of any indicator will be low unless the OR is very high. Therefore, only markers with an OR about 5 or more may have a sufficiently strong association to acidemia by itself to be considered as a ´pathological´ sign, indicating intervention.

This study showed that late, combined, and prolonged decelerations were strongly or moderately associated with acidemia at birth. This is consistent with previous studies [13], [14], [15]. We could also evaluate how the total number of decelerations during one hour of recording, and repetitivity influenced the risk of acidemia. As expected, the OR for acidemia increased with increasing number of decelerations. For late and combined decelerations, we also saw OR for repetitive decelerations that corresponded to OR for a higher number of decelerations. For variable decelerations > 60 s, no association between repetitive decelerations and acidemia was found, while a frequency of 10 or more showed a weak association with acidemia. Since the confidence intervals for these comparisons overlapped, this difference is probably coincidental. Moreover, we found a strong association between > 50 % total duration of decelerations for 30 min and acidemia at birth.

Previous studies have shown varying results regarding variable decelerations. Low et al. found that variable and early decelerations were not associated to asphyxia when the last 4 hours before birth was examined [14]. Holzmann et al. found that variable decelerations > 60 s increased the risk of elevated fetal scalp blood lactate, particularly in combination with tachycardia [13]. We found a weak relationship between variable decelerations > 60 s and acidemia, that remained also among traces with normal baseline and variability.

Since the NICE guidelines assess the risk of hypoxia in case of variable decelerations based on several different concerning characteristics [2], we performed a subanalysis of these and other characteristics for variable decelerations > 60 s. Only one characteristic, absent variability within the decelerations, showed a significant association to acidemia with OR 5.8 compared to those with variable decelerations > 60 s without this characteristic. Given the small number of recordings with decelerations with overshoot, loss of shouldering, absent variability within the deceleration and baseline > 160 bpm or variability < 5 bpm between the decelerations, these estimated ORs should be interpreted cautiously. Cahill et al. investigated the association between shoulders, overshoots, slow returns and variability in variable decelerations and did not find any association with metabolic acidemia [16]. Hamilton et al. reported an association to acidemia for variable decelerations with prolonged duration > 120 seconds, loss of internal variability or with two “sixties” criteria present: depth > 60 bpm or more, nadir < 60 bpm, or duration > 60 s or longer [17]. Martí Gamboa et al. reported an increased risk of acidemia for variable decelerations with "slow return" or loss of moderate variability within decelerations [18].

We considered it of interest to evaluate the association of NICE-22 and SWE-17 criteria for the assessment of variable decelerations as suspicious or pathological and acidemia at birth. Repetitive variable decelerations with any concerning characteristics (NICE-22) showed only a weak association with acidemia both for durations over and under 30 min. Variable decelerations > 60 s with baseline > 160 bpm or variability < 5 bpm (pathological in SWE-17) were also weakly associated with acidemia.

The criterion “50 % below baseline during 30 min” was associated with a high OR for acidemia without considering of the type of decelerations behind. None of current guidelines include this as a suspicious or pathological pattern. Considering our result, and previous studies [19], [20], this criterion might be useful when evaluating CTG in labour.

In the first stage of labour, late, combined, prolonged decelerations > 5 min, and variable decelerations > 60 s were rarely seen in controls and strongly associated with acidemia. This finding may indicate that the threshold for intervention should be lower in the first stage of labour, and that the only decelerations that should be accepted as a normal pattern in the first stage are early uniform decelerations and variable decelerations < 60 s.

To our knowledge, this study is the first to assess the associations between acidemia at birth and characteristics and numbers of different deceleration in such detail in a material including a relatively large number of traces from neonates with acidemia. Limitations are that the size of the material does not allow firm conclusions about fetal heart rate patterns that are infrequent also in cases with acidemia and that only the last hour of CTG recordings were analysed. A scientific limit, but ethical prerequisite, is that, like most studies of CTG, we did not study the natural course for fetuses with different heart rate patterns; interventions had been undertaken when patterns were recognized as pathological.

We consider that the results of the present study may be useful in endeavors to increase the diagnostic precision of CTG interpretation templates.

## 6 Conclusion

Intrapartum fetal heart rate patterns with repetitive late or combined decelerations, three or more 3–5 min prolonged decelerations or one deceleration prolonged for > 5 min entail a strongly increased risk for acidemia at birth. An equally elevated risk exists if the fetal heart rate is below the baseline more than 50 % of the time for 30 min. This variable may be valuable to include when assessing CTG during labour.

## Ethical approval

Ethical approval was obtained from the Regional Ethical Review Board in Lund, Dnr 2016/371, 24 May 2016.

## Funding

The study is part of a project supported by research grants from the Public Health Care Region Skåne and LÖF, the Swedish Patient Insurance Company.

## CRediT authorship contribution statement

**Fogelberg Maria:** Writing – original draft, Visualization, Methodology, Investigation, Formal analysis. **Dahlbäck Charlotte:** Writing – review & editing, Investigation. **Ekengård Frida:** Writing – review & editing, Resources, Investigation. **Rickle Gisela:** Writing – review & editing, Investigation. **Herbst Andreas:** Writing – review & editing, Visualization, Project administration, Methodology, Investigation, Funding acquisition, Formal analysis, Conceptualization.

## Declaration of Competing Interest

The authors declare that they have no known competing financial interests or personal relationships that could have appeared to influence the work reported in this paper.

## Data Availability

We do not have approved permission for data sharing from the ethics authority and therefore regrettably cannot share data on request.
